# H3K4me3‐Mediated FOXJ2/SLAMF8 Axis Aggravates Thrombosis and Inflammation in β2GPI*/*Anti‐β2GPI‐Treated Monocytes

**DOI:** 10.1002/advs.202309140

**Published:** 2024-04-19

**Authors:** Yuan Tan, Jiao Qiao, Shuo Yang, Hongchao Liu, Qingchen Wang, Qi Liu, Weimin Feng, Liyan Cui

**Affiliations:** ^1^ Institute of Medical Technology Peking University Health Science Center Beijing 100191 China; ^2^ Department of Laboratory Medicine Peking University Third Hospital Beijing 100191 China; ^3^ Core Unit of National Clinical Research Center for Laboratory Medicine Peking University Third Hospital Beijing 100191 China

**Keywords:** APS, epigenetics, FOXJ2, H3K4me3, inflammation, thrombosis

## Abstract

Antiphospholipid syndrome (APS) is characterized by thrombus formation, poor pregnancy outcomes, and a proinflammatory response. H3K4me3‐related monocytes activation are key regulators of APS pathogenesis. Therefore, H3K4me3 CUT&Tag and ATAC‐seq are performed to examine the epigenetic profiles. The results indicate that the H3K4me3 signal and chromatin accessibility at the *FOXJ2* promoter are enhanced in an in vitro monocyte model by stimulation with β2GPI*/*anti‐β2GPI, which mimics APS, and decreases after OICR‐9429 administration. Furthermore, FOXJ2 is highly expressed in patients with primary APS (PAPS) and is the highest in patients with triple‐positive antiphospholipid antibodies (aPLs). Mechanistically, FOXJ2 directly binds to the *SLAMF8* promoter and activates SLAMF8 transcription. SLAMF8 further interacts with TREM1 to stimulate TLR4/NF‐κB signaling and prohibit autophagy. Knockdown of FOXJ2, SLAMF8, or TREM1 blocks TLR4/NF‐κB and provokes autophagy, subsequently inhibiting the release of inflammatory and thrombotic indicators. A mouse model of vascular APS is established via β2GPI intraperitoneal injection, and the results suggest that OICR‐9429 administration attenuates the inflammatory response and thrombus formation by inactivating FOXJ2/SLAMF8/TREM1 signaling. These findings highlight the overexpression of H3K4me3‐mediated FOXJ2 in APS, which consequently accelerates APS pathogenesis by triggering inflammation and thrombosis via boosting the SLAMF8/TREM1 axis. Therefore, OICR‐9429 is a promising candidate drug for APS therapy.

## Graphical abstract

H3K4me3 signaling and chromatin accessibility at the *FOXJ2* promoter are enhanced in an in vitro monocyte model mimicking APS, and FOXJ2 is highly expressed in patients with primary APS. FOXJ2 overexpression activates SLAMF8/TREM1‐mediated inflammation and thrombosis by directly binding to the *SLAMF8* promoter. OICR‐9429 administration attenuates APS pathogenesis in vivo and in vitro.

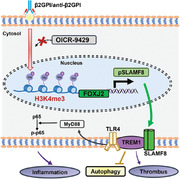

## 1 Introduction

Antiphospholipid syndrome (APS) is a heterogeneous disease characterized by autoimmune disorders, chronic inflammation, venous or arterial thrombosis, adverse pregnancy outcomes (APOs), and the presence of antiphospholipid antibodies (aPLs).^[^ ^1^ ^]^ aPLs are autoantibodies with a heterogeneous profile and include anti‐beta2‐glycoprotein I (anti‐β2GPI), anticardiolipin antibody (aCL), and lupus anticoagulant (LAC).^[^ ^2^ ^]^ These autoantibodies activate endothelial cells (ECs), monocytes, and platelets, contributing to the activation of hypercoagulable and hyperinflammatory states in APS.^[^ ^3^ ^]^ Current therapeutic options for APS are limited, and long‐term anticoagulation therapy is commonly used to prevent recurrent thrombosis. However, this is unfavorable for patients who have not yet suffered from thrombosis but have aPLs.^[^ ^4^ ^]^ Thus, the molecular mechanisms of APS development are worth exploring and would provide great opportunities for advanced treatment of APS.

Several studies have implicated epigenetic mechanisms in autoimmune disease pathogenesis. Epigenetic changes mainly involve histone modifications, non‐coding RNAs (ncRNAs), and DNA methylation, which regulate chromatin remodeling and downstream genes expression.^[^ ^5^ ^]^ Trimethylation of histone H3K4 (H3K4me3), a major histone modification mark that is strongly associated with active transcription, is mostly found around transcription start sites (TSSs).^[^ ^6^, ^7^ ^]^ In monocytes from patients with systemic sclerosis (SS), H3K4me3‐mediated gene alterations are enriched in immune, interferon (IFN), and antiviral pathways by providing binding sites for interferon regulatory factors (IRF) and signal transducer and activator of transcription (STAT) transcription factors in their promoters.^[^ ^8^ ^]^ In addition, H3K4me3 has been linked to the activation of the specificity protein 1 (SP1), activating transcription factor 3 (ATF3), and high mobility group A1 (HMGA1) promoters in T cells, B cells, and monocytes of patients with systemic lupus erythematosus (SLE) and is involved in SLE pathogenesis and progression.^[^ ^9^ ^]^ A previous study found elevated H3K4me3 signals at the matrix metalloproteinase (MMP)−1, −3, −9, and −13 promoters in synovial fibroblasts of patients with rheumatoid arthritis (RA), and upregulated MMPs are potential biomarkers of disease activity.^[^ ^10^ ^]^ OICR‐9429, a high‐affinity small‐molecule compound, potently suppresses the trimethylation of histone H3K4.^[^ ^11^, ^12^ ^]^ Nevertheless, the role of OICR‐9429 in inhibiting H3K4me3 and whether it can be used for the treatment of APS remain to be further investigated.

The β2GPI*/*anti‐β2GPI complex binds and activates monocytes.^[^ ^13^ ^]^ Activated monocytes cause APS development by inducing proinflammatory and prothrombotic responses,^[^ ^14^ ^]^ which might be attributed to epigenetic abnormalities of monocytes.^[^ ^13^, ^15^ ^]^ For example, DNA methylation at the interleukin (IL)−8 promoter and first intron of tissue factor 3 (TF3) increases at 4 h in a β2GPI*/*anti‐β2GPI‐stimulated monocyte model that mimics APS, followed by a decrease at 6 h and a return to basal levels at 24 h post‐stimulus. Changes in the dynamic methylation of IL‐8 and TF3 cause transcriptional activation.^[^ ^13^ ^]^ Significantly, these epigenetically altered genes are responsible for aggravating coagulation formation, proinflammatory reactions, and poor pregnancy outcomes in patients with APS.

Considering the vital role that the epigenetic distortion of monocytes plays in APS, H3K4me3 CUT&Tag and ATAC‐seq were designed to examine H3K4me3 signaling and chromatin accessibility at the whole‐genome level in an in vitro monocyte model mimicking APS. Integrative analysis identified augmented H3K4me3 signaling and chromatin accessibility at the forkhead box J2 (*FOXJ2*) promoter. Moreover, FOXJ2 was highly expressed in patients with primary APS (PAPS) and in an in vivo mouse model mimicking APS. The level of FOXJ2 was highest in patients with PAPS with triple‐positive aPLs, indicating the pivotal function of FOXJ2 in APS pathogenesis. Therefore, a series of experiments was conducted to investigate the corresponding molecular mechanisms and clarify whether OICR‐9429 can alleviate APS pathogenesis, which could provide a novel therapeutic target for patients with APS.

## 2 Results

### 2.1 Epigenetic Profiles of the In Vitro Monocyte Model Mimic APS

**Figure 1:**
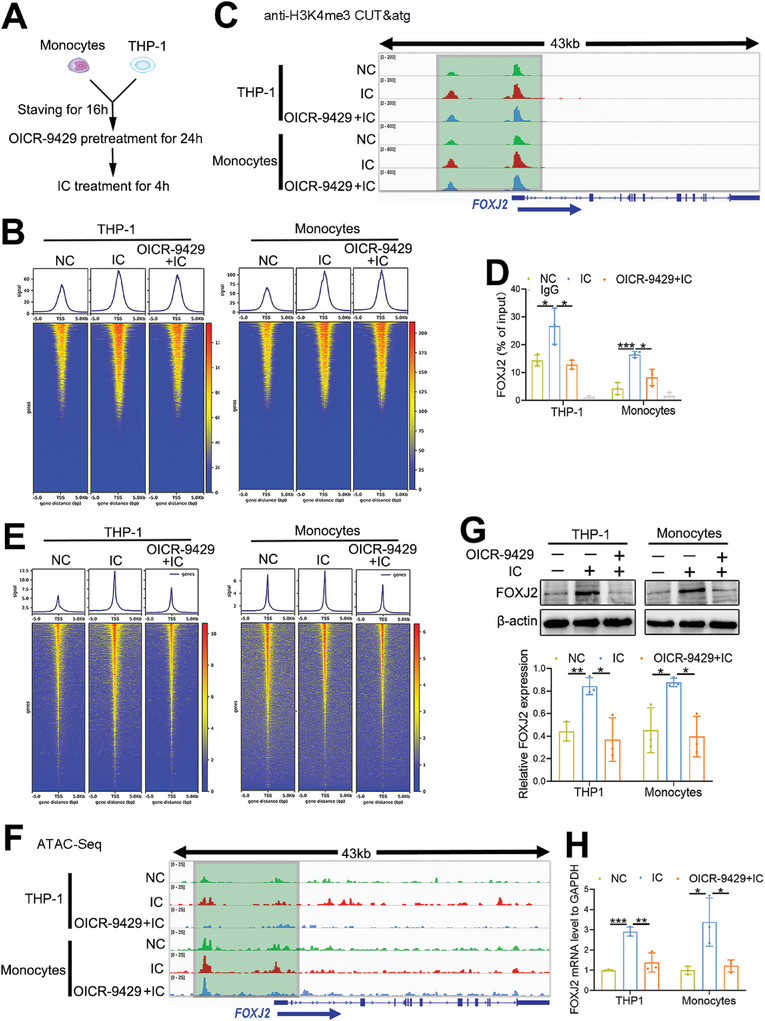
Epigenetic profiles in an in vitro monocyte model mimicking APS. A) Workflow of IC treatment and OICR‐9424 exposure in ex vivo monocytes or THP‐1 cells model partially mimic APS. B) Heatmaps of H3K4me3 CUT&Tag‐Seq at the whole genome level in an in vitro APS model using THP‐1 cells and monocytes, respectively. C) The relative enrichment levels of H3K4me3 at the promoter of *FOXJ2* are visualized using IGV. D) H3K4me3 CUT&Tag‐qPCR tested the relative enrichment levels of H3K4me3 at the *FOXJ2* promoter in an in vitro APS model of THP‐1 cells and monocytes, respectively. E) Heatmaps of chromatin accessibility at the whole‐genome level from ATAC‐seq analysis of an in vitro APS model of THP‐1 cells and monocytes, respectively. F) The chromatin accessibility at the *FOXJ2* promoter is displayed using IGV. G) The level of FOXJ2 in vitro APS model is detected using western blotting. H) The expression of FOXJ2 in the in vitro APS model is determined using RT‐qPCR. Data are expressed as the mean ± SEM of three independent experiments. IGV, Integrative Genomics Viewer; *P < 0.05; **P < 0.01; ***P < 0.001.

H3K4me3 serves as a canonical epigenetic mark and represents transcription initiation and elongation.^[^ ^16^, ^17^ ^]^ OICR‐9429, an inhibitor targeting the MLL1‐WDR5 interaction, can repress the H3K4me3 mark and related gene transcription.^[^ ^11^, ^12^ ^]^ In order to explore H3K4me3‐mediated APS pathogenesis, we first built an in vitro APS model via stimulating monocytes or THP‐1 cells with the β2GPI/anti‐β2GPI IC. Samples were divided into NC, IC, and OICR‐9429+IC groups for both monocytes and THP‐1 cells (**Figure** **1**A). H3K4me3 CUT&Tag‐seq was used to detect H3K4me3 signatures at the whole‐genome level in an in vitro monocyte model that mimicked APS. In line with the described canonical H3K4me3 pattern, the H3K4me3 signal was mainly enriched in the TSS region (Figure 1B). Moreover, the specific H3K4me3 signal at the *FOXJ2* promoter was increased in IC‐stimulated monocytes mimicking APS but was remarkably reduced upon treatment with OICR‐9429 (Figure 1C). CUT&Tag‐qPCR further indicated increased enrichment of H3K4me3 at the *FOXJ2* promoter in the in vitro APS model (Figure 1D).

Chromatin accessibility plays a crucial role in determining gene expression profiles by opening or closing accessibility. Open accessibility predisposes to autoimmune diseases by upregulating the expression of inflammatory genes.^[^ ^18^ ^]^ Therefore, this study utilized ATAC‐seq to test chromatin accessibility at the whole‐genome level and found that highly open accessibility was mainly located at the TSS site (Figure 1E). Moreover, chromatin accessibility at the *FOXJ2* promoter was specifically augmented in the in vitro APS model but was attenuated by OICR‐9429 treatment (Figure 1F).

FOXJ2, as a member of forkhead/HNF3 family, has the capability to maintain LPS‐induced production of inflammatory cytokines, such as IL‐6 and tumor necrosis factor (TNF)‐α.^[^ ^19^ ^]^ Therefore, FOXJ2 was the focus of subsequent experiments. FOXJ2 was overexpressed in the IC‐stimulated APS model, whereas its expression was dramatically reduced upon exposure to OICR‐9429 (Figure 1G,H). Therefore, IC administration in the in vitro APS model specifically strengthened the H3K4me3 signal and chromatin accessibility at the *FOXJ2* promoter, which facilitated FOXJ2 expression in APS.

### 2.2 Elevated FOXJ2 Expression in Patients with PAPS

**Figure 2:**
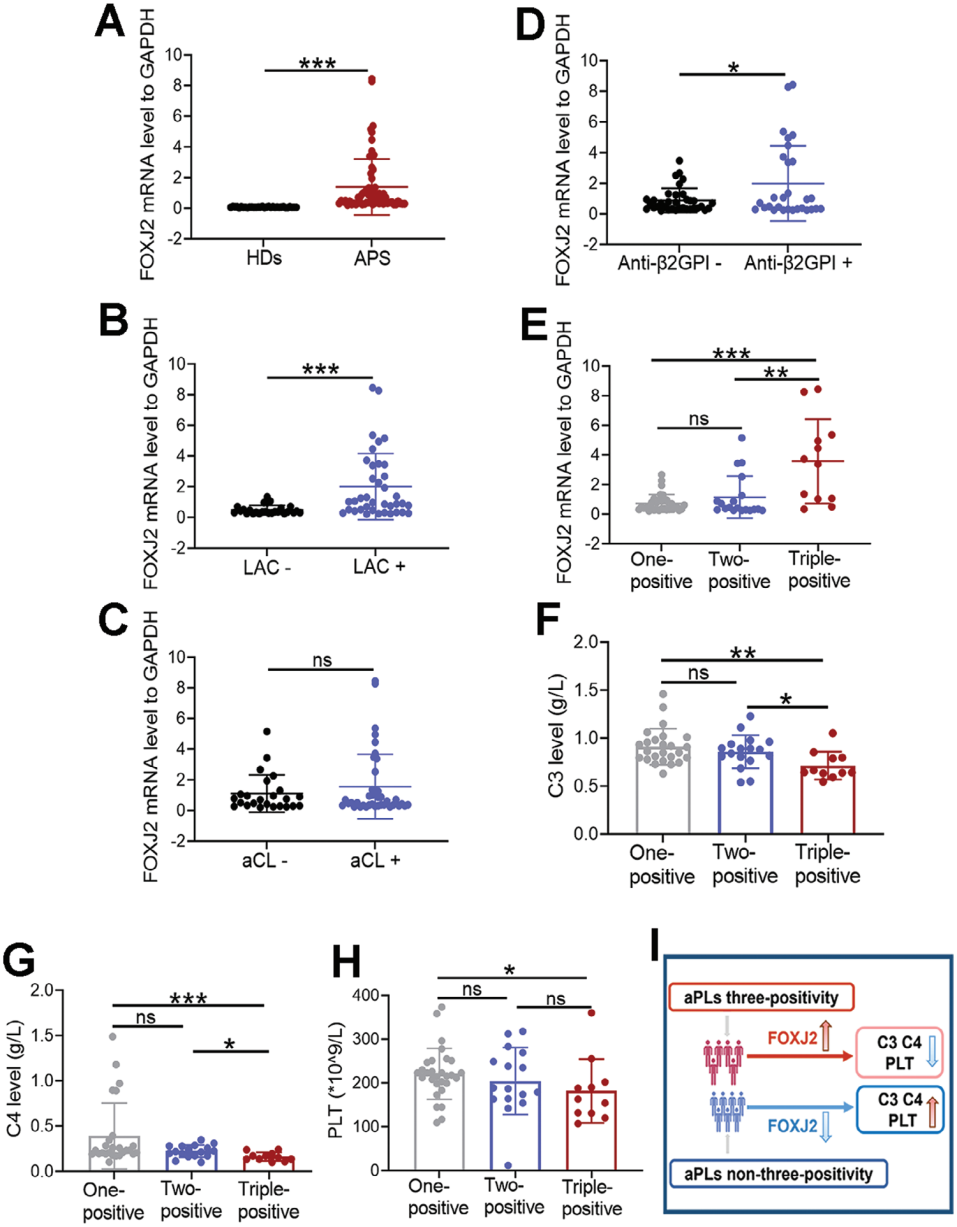
Elevated FOXJ2 expression in patients with PAPS. A) Relative FOXJ2 mRNA level in patients with PAPS and HDs. B) Relative FOXJ2 mRNA level in LAC‐positive and ‐negative patients with PAPS. C) Relative FOXJ2 mRNA level in aCL‐positive and ‐negative patients with PAPS. D) Relative FOXJ2 mRNA level in anti‐β2GPI‐positive and ‐negative patients with PAPS. E) The expression level of FOXJ2 in patients with PAPS and triple‐, double‐, and single‐positive aPLs. F) C3 levels in single‐, double‐, and triple‐positive patients with PAPS. G) C4 levels in single‐, double‐, and triple‐positive patients with PAPS. H) PLT levels in single‐, double‐, and triple‐positive patients with PAPS. I) The correlation of FOXJ2 with the aPL positive rate and the levels of C3, C4, and PLT. PAPS, primary APS; HDs, healthy donors; *P < 0.05; **P < 0.01; ***P < 0.001.

Since FOXJ2 was overexpressed in the in vitro APS model, we explored the clinical relationship between FOXJ2 and patients with PAPS. Primary monocytes from 64 patients with PAPS and 32 HDs were collected to examine the expression of FOXJ2, and their clinical baseline data were obtained (Data S1, Supporting Information). FOXJ2 expression was higher in patients with PAPS than in HDs (**Figure** **2**A). The mRNA level of FOXJ2 was higher in aPL‐positive patients with PAPS than in aPL‐negative patients (Figure 2B–D) and was the highest among patients with PAPS with triple‐positive aPLs (Figure 2E). Notably, PLT and complement C3 and C4 levels were associated with APS progression. Among the patients with PAPS who had laboratory data for C3, C4, and PLT, the levels of C3, C4, and PLT showed a decreasing tendency in patients with PAPS with triple‐positive aPLs compared with those of patients with single‐ or double‐positive aPLs (Figure 2F–H). However, the difference was only significant between aCL‐positive and aCL‐negative patients with PAPS (**Table** **1**). The positivity rate of other aPLs did not correlate with a history of thrombosis or adverse pregnancy delivery (Table 1). The above findings indicated that the expression of FOXJ2 was increased in patients with PAPS and the level of FOXJ2 positively correlated with the positive rate of aPLs (Figure 2I). Moreover, aPLs may have affected the levels of C3, C4, and PLT.

**Table 1:** The association of clinical findings with aPLs in patients with PAPS.

Clinical Features	LAC		P	aCL		P	*Anti*‐β2GPI		P
	+	−		+	−		+	−	
Number	38	26		40	24		29	35	
Median age in years	35.00(30.75, 37. 25)	36.15 ± 0.96	0.32	35.00(32.00, 38.75)	34.58 ± 1.29	0.36	34.00(32.00, 37.50)	35.94 ± 1.02	0.49
History of thrombosis	12	3	0.12	7	8	0.15	5	10	0.29
History of adverse pregnancy delivery	32	25	0.27	36	21	0.92	26	31	0.79
PLT (*10^9^/L)	202.7 ± 11.31	219.1 ± 14.87	0.39	194.10 ± 11.80	230.90 ± 12.68	0.04	210.50 ± 15.52	215.50(184, 242.8)	0.62
C3 (g L^−1^)	0.82 ± 0.03	0.85(0.80, 0.96)	0.23	0.81 ± 0.03	0.93 ± 0.05	0.03	0.83 ± 0.05	0.85(0.78, 0.96)	0.27
C4 (g L^−1^)	0.21(0.17, 0.26)	0. 23 ± 0.02	0.61	0.20 ± 0.01	0.25(0.20, 0.75)	0.01	0.21 ± 0.01	0.22(0.19, 0.28)	0.06

### 2.3 FOXJ2 Accelerates Inflammation and Thrombosis by Transcriptionally Activating SLAMF8 In Vitro

**Figure 3:**
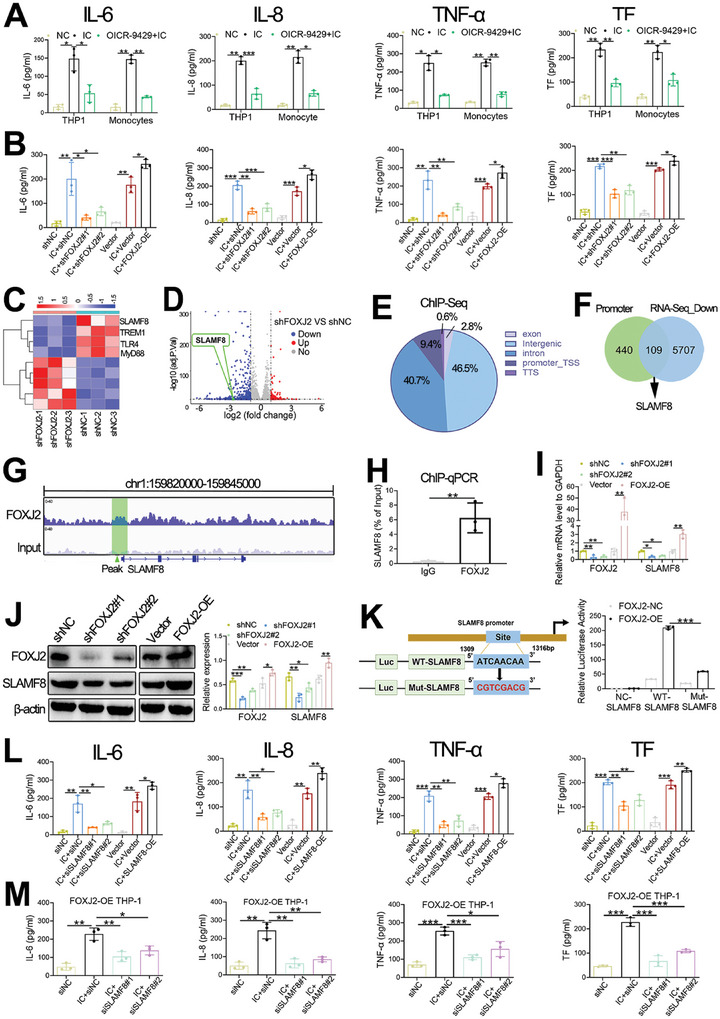
FOXJ2 accelerated inflammation and thrombosis by transcriptionally activating SLAMF8 in vitro. A) ELISA is utilized to determine the concentrations of IL‐6, IL‐8, TNF‐α, and TF in an IC‐induced APS model and their levels with OICR‐9429 administration. B) The effect of FOXJ2 knockdown or overexpression on the secretion of IL‐6, IL‐8, TNF‐α, and TF is examined using ELISA. C) A heatmap shows the normalized expression of several deregulated genes between shNC and shFOXJ2‐THP‐1 cells. D) Volcano plot of differentially expressed genes between shNC and shFOXJ2‐THP‐1 cells. E) The distribution of FOXJ2 ChIP‐Seq peaks. F) The integrative analysis between FOXJ2 ChIP‐Seq and RNA‐Seq. G) IGV representation of anti‐FOXJ2 ChIP‐Seq at the *SLAMF8* promoter in THP‐1 cells. H) ChIP‐qPCR indicates the relative enrichment level of FOXJ2 within the *SLAMF8* promoter in THP‐1 cells, with control IgG as a negative control. I) mRNA levels of FOXJ2 and SLAMF8 in FOXJ2‐knockdown and FOXJ2‐OE THP‐1 cells are detected with RT‐qPCR. J) The protein levels of FOXJ2 and SLAMF8 in FOXJ2‐knockdown and FOXJ2‐OE THP‐1 cells, with β‐actin as a loading control. K) Schematic representation of the FOXJ2 target sequence within the *SLAMF8* promoter; the luciferase activities of WT‐SLAMF8 and Mut‐SLAMF8 are measured after cotransfection with FOXJ2‐OE or FOXJ2‐NC. L) IL‐6, IL‐8, TNF‐α, and TF levels in siSLAMF8 and SLAMF8‐OE THP‐1 cells are detected using an ELISA. M) IL‐6, IL‐8, TNF‐α, and TF concentrations in siSLAMF8 and FOXJ2‐OE cotransfected THP‐1 cells are detected using an ELISA. Data are expressed as the mean ± SEM of three independent experiments. TSS, transcription start site. *P < 0.05; **P < 0.01; ***P < 0.001.

After confirming the elevated FOXJ2 expression in patients with PAPS, we next examined the effect of FOXJ2 on the inflammatory response and thrombotic formation in APS. ELISA quantification was used to determine the levels of related inflammatory and thrombotic cytokines. In particular, the levels of IL‐6, IL‐8, TNF‐α, and tissue factor (TF) were increased in the IC group, while the use of OICR‐9429 limited their production (**Figure** **3**A). Further, knockdown of FOXJ2 in THP‐1 cells dramatically restrained IC‐induced IL‐6, IL‐8, TNF‐α, and TF secretion, whereas FOXJ2 overexpression promoted their secretion (Figure 3B). Therefore, overexpressed FOXJ2 facilitates APS progression by stimulating IL‐6, IL‐8, TNF‐α, and TF production, and OICR‐9429 may mitigate APS pathogenesis by downregulating FOXJ2.

To further explore the underlying mechanisms of FOXJ2 in APS, RNA‐seq was used to screen for FOXJ2‐modulated genes. Significantly, the results showed 549 downregulated and 123 upregulated genes in FOXJ2‐knockdown THP‐1 cells (Figure 3C,D; Data S2, Supporting Information). Meanwhile, anti‐FOXJ2 ChIP‐Seq was conducted, and the results showed that 9.4% of the FOXJ2 peaks were located on the promoters (Figure 3E). Integrative analysis confirmed that 109 promoter peak‐annotated genes were downregulated after FOXJ2 downregulation, including guanosine monophosphate reductase (GMPR), dynein axonemal heavy chain 8 (DNAH8), mast cell‐expressed membrane protein1 (MCEMP1), and signaling lymphocyte activating molecule family member 8 (SLAMF8) (Figure 3F). The relationship between GMPR, DNAH8, MCEMP1, and autoimmune diseases has not yet been reported. SLAMF8 is an essential surface receptor that is highly expressed on monocytes and has been implicated in the progression of autoimmune diseases such as RA.^[^ ^20^ ^]^ Moreover, FOXJ2 regulated SLAMF8 by directly binding to the *SLAMF8* promoter (Figure 3G; Data S3, Supporting Information), with a 21.6‐fold increase in the binding of FOXJ2 to the *SLAMF8* promoter compared with the nonspecific binding of IgG to the *SLAMF8* promoter (Figure 3H). RT‐qPCR and western blotting revealed the downregulated SLAMF8 after FOXJ2 knockdown and the upregulated SLAMF8 after FOXJ2 overexpression (Figure 3I,J). In addition, the luciferase activity reporter assay further determined that the mutated promoter reporter resulted in decreased luciferase reporter activity for the *SLAMF8* promoter (Figure 3K). Taken together, these results indicate that FOXJ2 directly induces *SLAMF8* promoter transcription.

To explore whether the protective effect of SLAMF8 inhibition on APS pathogenesis is associated with reduced inflammation and thrombosis, we compared the levels of inflammatory and thrombotic indicators in SLAMF8‐OE and SLAMF8‐knockdown THP‐1 cells. Qualification results showed increased production of IL‐6, IL‐8, TNF‐α, and TF in SLAMF8‐OE THP‐1 cells and reduced production in SLAMF8‐knockdown THP‐1 cells (Figure 3L). We then cotransfected siSLAMF8 and FOXJ2‐OE into THP‐1 cells to conduct rescue experiments. The data showed that SLAMF8 knockdown attenuated the production of IL‐6, IL‐8, TNF‐α, and TF in FOXJ2‐OE THP‐1 cells after IC stimulation (Figure 3M). Thus, FOXJ2 plays an important role in IC‐induced inflammation and thrombosis via SLAMF8 overexpression.

### 2.4 SLAMF8 Directly Interacts with TREM1 to Modulate Thrombosis and Inflammatory Responses Related to TLR4/NF‐κB

**Figure 4:**
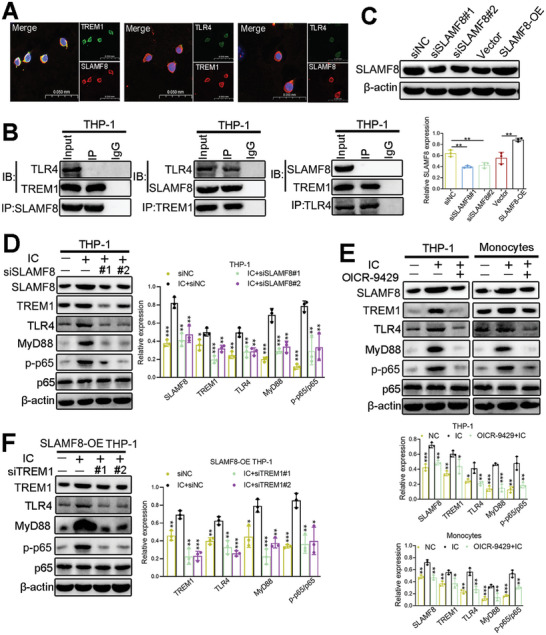
SLAMF8/TREM1 promotes thrombosis and inflammatory response related to TLR4/NF‐κB signaling. A) Double‐labeled immunofluorescence detects the colocation between SLAMF8 and TREM1 or TLR4 (40X). B) Verification of the interaction between SLAMF8 and TREM1 or TLR4 by co‐IP assay. C) The efficiency of SLAMF8 knockdown and overexpression is detected by western blotting. D) The expression of SLAMF8, TREM1, TLR4, MyD88, and p‐p65 NF‐κB in siSLAMF8‐transfected THP‐1 cells after IC stimuli. E) Quantification of SLAMF8, TREM1, TLR4, MyD88, and p‐p65 NF‐κB is detected in IC‐treated and OICR‐9429 plus IC‐treated cells. F) The expression of TREM1, TLR4, MyD88, and p‐p65 NF‐κB in THP‐1 cells cotransfected with siTREM1 and SLAMF8‐OE, with β‐actin as a loading control. Data are expressed as the mean ± SEM of three independent experiments. *P < 0.05; **P < 0.01; ***P < 0.001.

SLAMF8 acts as a novel regulator of macrophage‐mediated inflammation by activating TLR4/NF‐κB signaling but does not depend on the direct regulation of TLR4.^[^ ^20^, ^21^ ^]^ Triggering receptor expressed on myeloid cells 1 (TREM1) is an active receptor expressed on human monocytes and macrophages that facilitates the LPS‐induced inflammatory response by directly binding to TLR2 and TLR4.^[^ ^22^ ^]^ Considering that TERM1 was significantly downregulated in FOXJ2‐knockdown THP‐1 cells (Figure 3C), we hypothesized that SLAMF8 participates in the TLR4/NF‐κB axis by stabilizing TREM1 expression through molecular interaction. To validate this hypothesis, we performed co‐IP and double‐labeled immunofluorescence assays. Double‐labeled immunofluorescence showed that SLAMF8, TREM1, and TLR4 were mainly expressed on the cell membrane in THP‐1 cells (**Figure** **4**A). Nevertheless, co‐IP data indicated that SLAMF8 interacted with TREM1 but not with TLR4 and that TREM1 co‐immunoprecipitated with TLR4 (Figure 4B).

Based on the above verifications, subsequent experiments examined whether SLAMF8 could affect the TREM1/TLR4/NF‐κB signaling pathway. First, SLAMF8‐OE and siSLAMF8 transfections were performed. Western blotting revealed that SLAMF8 expression was significantly increased in THP‐1 cells transfected with SLAMF8‐OE but decreased in cells transfected with siSLAMF8 (Figure 4C). Further, results suggested that SLAMF8 knockdown markedly repressed the expression of TREM1, TLR4, MyD88, and phosphorylated‐p65 (p‐p65) NF‐κB in the IC group (Figure 4D). In addition, OICR‐9429 administration suppressed IC‐enhanced TREM1/TLR4/NF‐κB signaling (Figure 4E). To further confirm that TREM1 intervened in SLAMF8‐activated TLR4/NF‐κB signaling, we transfected siTREM1 into SLAMF8‐OE THP‐1 cells and found that TREM1 knockdown suppressed SLAMF8‐stimulated TLR4/NF‐κB signaling (Figure 4F). The above results verified that SLAMF8 activates TLR4/NF‐κB‐dependent inflammation by positively modulating TREM1 expression, resulting in APS deterioration.

### 2.5 SLAMF8/TREM1 Complex Blocks Autophagy in the In Vitro APS Model

**Figure 5:**
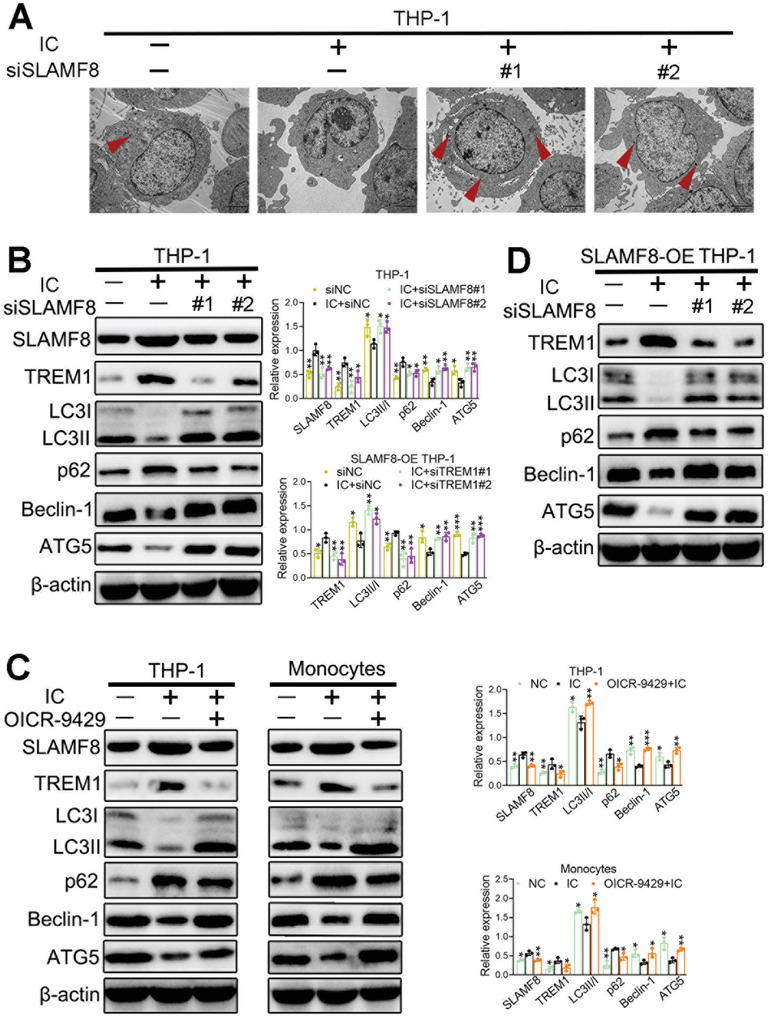
SLAMF8/TREM1 complex blocks autophagy in an in vitro APS model. A) Representative TEM images of autophagic vacuoles (red arrows) in THP‐1 cells. B) The expression of SLAMF8, TREM1, LC3II/I, ATG5, Beclin‐1, and p62/SQSTM1 is detected in siSLAMF8‐transfected THP‐1 cells after IC stimuli. C) Quantification of SLAMF8, TREM1, LC3II/I, ATG5, Beclin‐1, and p62/SQSTM1 in IC‐treated and OICR‐9429 plus IC‐treated cells. D) The expression of TREM1, LC3II/I, ATG5, Beclin‐1, and p62/SQSTM1 in THP‐1 cells cotransfected with siTREM1 and SLAMF8‐OE, with β‐actin as a loading control. Data are expressed as the mean±SEM of three independent experiments. TEM, transmission electron microscopy; *P < 0.05; **P < 0.01; ***P < 0.001.

The β2GPI/anti‐β2GPI complex impairs autophagy, and deficient autophagy plays proinflammatory and prothrombotic roles in APS development.^[^ ^23^, ^24^ ^]^ Moreover, SLAMF8/TREM1 exerts crucial functions in restraining the autophagic response.^[^ ^25^, ^26^ ^]^ To investigate whether SLAMF8/TREM1 participates in APS pathogenesis by autophagy, cells were subjected to TEM to visualize the formation of autophagic vacuoles. As shown in **Figure** **5**A, the number of autophagic vacuoles significantly decreased upon IC stimulation but increased after SLAMF8 knockdown, indicating the protective effect of SLAMF8 deletion on autophagy. We also examined the expression of related autophagic molecules. In the IC group, the expression levels of LC3II/I, ATG5, and Beclin‐1 were reduced, whereas those of p62/SQSTM1, TREM1, and SLAMF8 were increased, indicating impaired autophagy (Figure 5B,C). Significantly, SLAMF8 deletion or OICR‐9429 administration rescued their expression, indicating that autophagy was provoked (Figure 5B,C). Additionally, TREM1 knockdown rescued SLAMF8‐injured autophagy after IC stimulation (Figure 5D). Taken together, SLAMF8 impairs autophagy by positively regulating TREM1 expression, further exacerbating the inflammatory response and APS pathogenesis.

### 2.6 Intervention of TREM1 Rescues FOXJ2/SLAMF8‐Induced Inflammation and Thrombosis In Vitro

**Figure 6:**
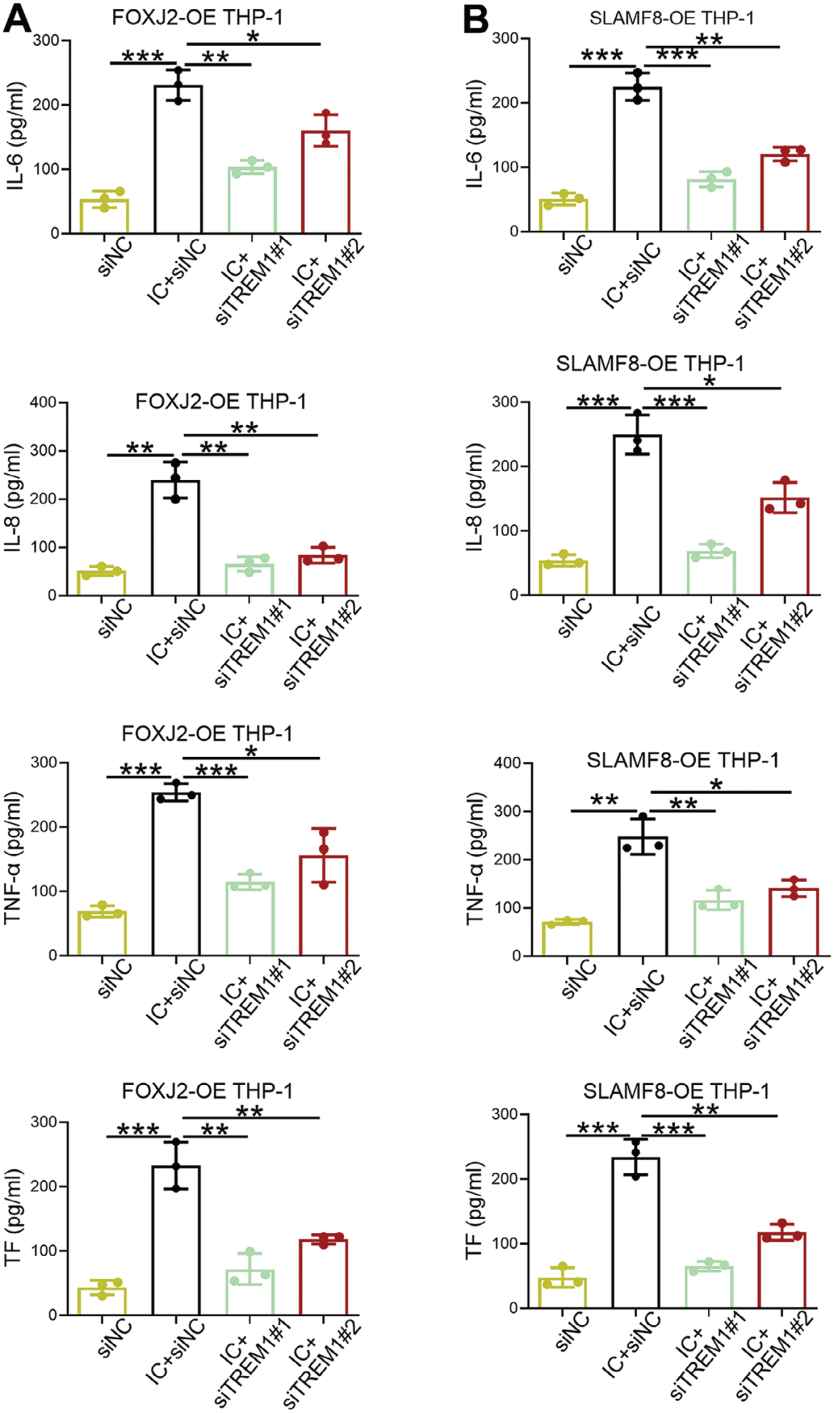
TREM1 intervention rescues SLAMF8‐ or FOXJ2‐induced inflammation and thrombosis in APS. A) Production levels of IL‐6, IL‐8, TNF‐α, and TF in THP‐1 cells cotransfected with siTREM1 and FOXJ2‐OE are detected using an ELISA. B) The concentrations of IL‐6, IL‐8, TNF‐α, and TF in THP‐1 cells cotransfected with siTREM1 and SLAMF8‐OE are detected using an ELISA. Data are expressed as the mean ± SEM of three independent experiments. *P < 0.05; **P < 0.01; ***P < 0.001.

Based on the verification of the FOXJ2/SLAMF8/TREM1 pathway, we explored whether TREM1 deletion ameliorated SLAMF8‐ or FOXJ2‐mediated APS pathogenesis in vitro. Cells cotransfected with siTREM1 and SLAMF8‐OE or FOXJ2‐OE were established to test the concentrations of IL‐6, IL‐8, TNF‐α, and TF. Quantification ELISA results showed that TREM1 deletion reduced the secretion of IL‐6, IL‐8, TNF‐α, and TF in SLAMF8‐OE and FOXJ2‐OE THP‐1 cells after IC administration, suggesting that TREM1 knockdown alleviated SLAMF8‐ or FOXJ2‐induced proinflammatory and thrombotic cytokine secretion (**Figure** **6**A,B). Taken together, these results suggest that FOXJ2/SLAMF8 facilitates APS deterioration by increasing the protein level of TREM1.

### 2.7 FOXJ2 Promotes APS Pathogenesis by Stimulating the SLAMF8/TREM1 Pathway In Vivo

**Figure 7:**
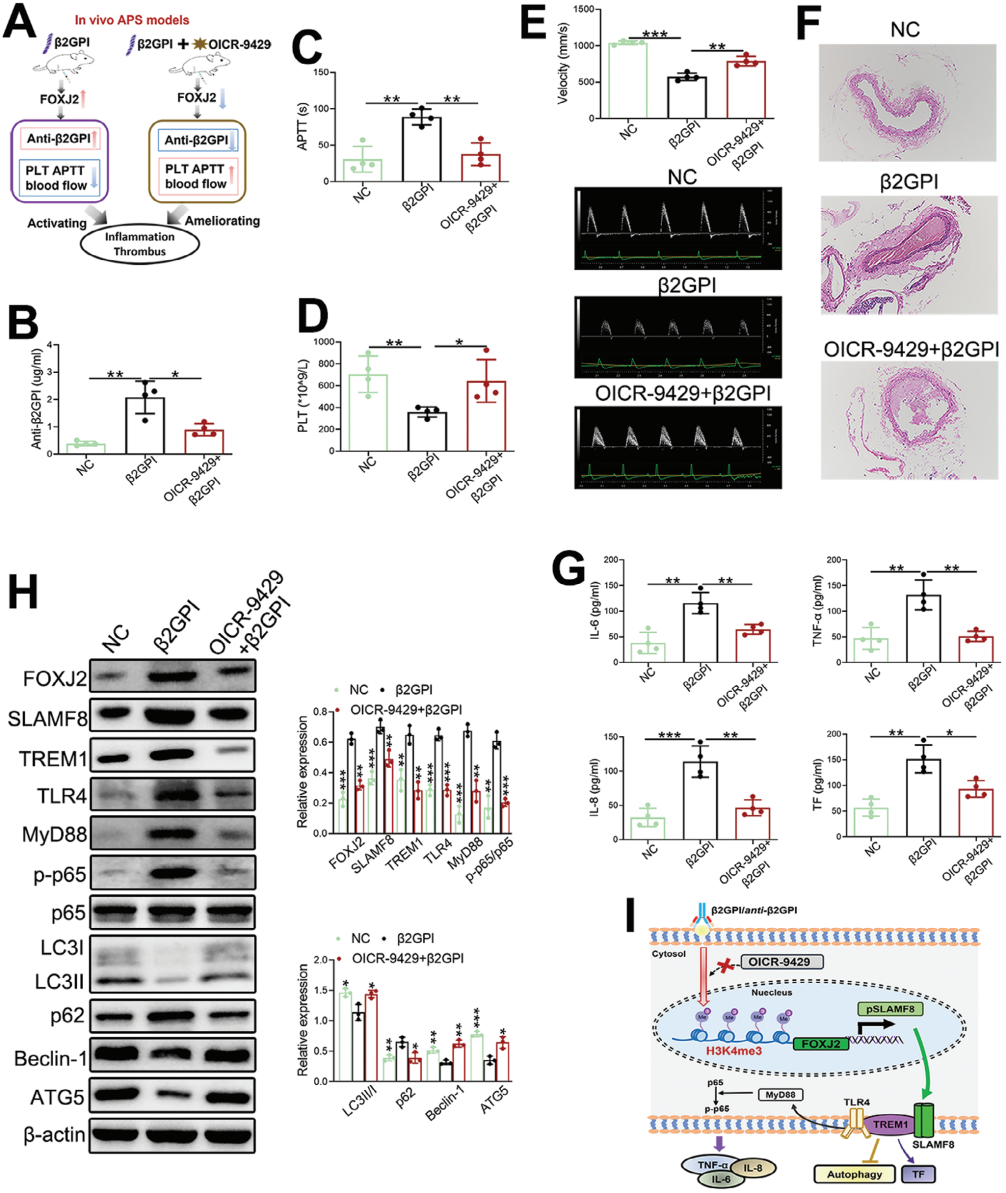
FOXJ2 promotes APS pathogenesis by stimulating the SLAMF8/TREM1 pathway in vivo. A) Experimental setup for β2GPI injection and OICR‐9429 administration in the in vivo mouse model of vascular APS (number of mice in each group = 4). B–D) Anti‐β2GPI levels, APTT, and PLT are detected in the NC, β2GPI, and OICR‐9429+β2GPI groups. E) Doppler analysis of the blood velocity of the ascending aorta. F) Thrombus size in the NC, β2GPI, and OICR‐9429+β2GPI groups (10X). G) The concentrations of IL‐6, IL‐8, TNF‐α, and TF are detected using an ELISA. H) The protein expression levels of FOXJ2, SLAMF8, TREM1, TLR4, MyD88, p‐p65/p65, LC3II/I, ATG5, Beclin‐1, and p62/SQSTM1 in bone marrow‐derived monocytes are detected using western blotting, with β‐actin as a loading control. I) The potential mechanisms of H3K4me3‐mediated FOXJ2 in APS pathogenesis. Data are expressed as the mean ± SEM of three independent experiments. NC, negative control; pSLAMF8, the promoter of SLAMF8; TF, tissue factor; *P < 0.05; **P < 0.01; ***P < 0.001.

In order to further confirm the in vivo function of FOXJ2, two groups of mice were used to establish a model of APS using intraperitoneal injections of 100 ug β2GPI thrice; one of β2GPI groups received OICR‐9429 for 7 consecutive days (**Figure** **7**A). On day 21, the β2GPI group had the increased anti‐β2GPI levels, indicating that the mouse model with APS was successfully established (Figure 7B). The β2GPI group had the longest APTT (Figure 7C), while PLT was reduced in the β2GPI group but increased under OICR‐9429 treatment (Figure 7D). On day 28, the β2GPI group had decreased ascending aortic blood velocity, which increased after OICR‐9429 treatment (Figure 7E). The thrombus size was greatly increased in the β2GPI group, and the use of OICR‐9429 remarkably decreased the thrombus formation (Figure 7F). Furthermore, the APS model had increased serum concentrations of IL‐6, IL‐8, TNF‐α, and TF that significantly decreased with OICR‐9429 administration (Figure 7G). In addition, western blot analysis validated the enhanced FOXJ2/SLAMF8/TREM1/TLR4 signaling and impaired autophagy in bone marrow‐derived monocytes from mice with APS, whereas the use of OICR‐9429 produced the opposite effect (Figure 7H). Therefore, FOXJ2‐mediated inflammation and thrombus formation are activated but autophagy is suppressed in a mouse model of APS, and OICR‐9429 may relieve APS pathogenesis by inactivating FOXJ2/SLAMF8/TREM1 signaling (Figure 7I).

## 3 Discussion

APS pathogenesis is partly orchestrated by epigenetic aberrance.^[^ ^6^, ^27^ ^]^ Monocytes play a key role in aPLs‐mediated APS pathogenesis by upregulating TF, vascular cell adhesion molecule‐1 (VCAM‐1), IL‐6, IL‐8, and TNF‐α expression.^[^ ^13^, ^28^ ^]^ Accumulating evidence suggests the therapeutic potential of epigenetics targeting monocytes. Therefore, this study sought evidence of epigenetic alterations in monocytes in APS.^[^ ^29^ ^]^ Considering the association of extended and enhanced H3K4me3 peaks with genes modulating immune responses,^[^ ^30^ ^]^ H3K4me3 CUT&Tag and ATAC‐seq were used to assess the profiles of H3K4me3 peaks and chromatin accessibility at the genome‐wide level, respectively. Specifically, the enhanced H3K4me3 peak and chromatin accessibility at the *FOXJ2* promoter were closely aligned with increased FOXJ2 transcription in patients with PAPS, as well as in an in vivo mouse model and an in vitro monocyte model mimicking APS. However, the mechanisms of FOXJ2 involvement in APS remain unclear.

FOXJ2 plays a crucial role in regulating the expression of immune response‐related genes through recognizing type A and B DNA sequences.^[^ ^31^, ^32^ ^]^ Type A sequences possess a core element that also exists in other forkhead factors, whereas type B sequences do not hold this core element. The frequency of type B sequences was lower than that of type A sequences in the site selection experiments; however, type A and B sequences have comparable affinities for fork head homologous X (FHX) sites.^[^ ^33^ ^]^ Previous studies have reported a positive association between FOXJ2 and the pathophysiology of cancers as well as autoimmune disorders.^[^ ^34^, ^35^, ^36^ ^]^ In this study, we identified the upregulation of FOXJ2 in APS for the first time, demonstrating the important role of FOXJ2 in monocytes for regulating APS pathogenesis. Considering the essential roles of activated ECs and platelets in APS, FOXJ2 might also epigenetically regulate the function of ECs and platelets during the progression of APS, which is worth investigating.

Furthermore, we identified the binding sites of FOXJ2 in the promoter region of *SLAMF8* and verified that SLAMF8 is a transcriptional target of FOXJ2 using ChIP‐seq and luciferase activity reporter assays. As a cell surface receptor, SLAMF8 is highly expressed upon exposure to proinflammatory stimuli.^[^ ^21^ ^]^ SLAMF8 upregulation has also been observed in autoimmune inflammation, such as RA.^[^ ^20^, ^37^ ^]^ Previous reports have demonstrated the important role of SLAMF8 in altering LPS‐mediated inflammation and autophagy by regulating mitogen‐activated protein kinase (MAPK) and TLR4 signaling.^[^ ^20^, ^21^ ^]^ On the other side, some studies have indicated that SLAMF8 attenuates the inflammation process in bacterial infected‐macrophages by inhibiting Nox2 activity through the PKC and PI3K pathways, these results might be involved in macrophage's microbicidal mechanisms based on bacterial infection‐induced models.^[^ ^38^, ^39^ ^]^ Thereby, different stimuli for constructing different disease models will trigger distinct pathological responses, which may lead to different or even conflicting results. Of importance, our studies confirmed that FOXJ2 participates in IC‐induced APS pathogenesis by positively modulating the expression of SLAMF8. Furthermore, the expression of SLAMF8 was increased in APS and SLAMF8 knockdown was demonstrated to ameliorate FOXJ2‐mediated inflammation and thrombosis in APS.

TREM and TREM‐like receptors are associated with inflammatory responses in both innate and adaptive immunity.^[^ ^40^ ^]^ Among these, TREM1 partakes in TLR4‐mediated inflammation and autophagy.^[^ ^26^, ^41^ ^]^ A study reported that TREM1 directly binds with TLR4 to boost TLR4/NF‐κB signaling,^[^ ^22^ ^]^ which plays an essential role in the pathogenesis of various autoimmune diseases, including RA, SLE, IBD, and type 1 diabetes (T1D).^[^ ^42^ ^]^ Most importantly, elevated TREM1 expression has been verified in patients with thrombotic PAPS and shows great potential for predicting thrombotic events and inflammatory activity.^[^ ^43^ ^]^ In addition, TLR4 exacerbates the pathogenesis of APS, and TLR4 deficiency prevents inflammation, thrombus formation, and TF activity in a murine model of APS.^[^ ^44^ ^]^ Taken together, both SLAMF8 and TREM1 are correlated with the amplification of TLR4 signaling. Therefore, we assessed the interaction of SLAMF8 and TREM1 with TLR4 and showed that SLAMF8 binds to TREM1 but not to TLR4, and TREM1 binds to TLR4. Moreover, SLAMF8 deletion inactivates TREM1/TLR4/NF‐κB signaling, and TREM1 knockdown abates the production of FOXJ2/SLAMF8‐induced inflammatory and thrombotic indicators. Of note, knockdown of SLAMF8 did not influence the TREM1 mRNA level (Figure S2A, Supporting Information), but repressed the protein expression of TREM1 (Figure S2B, Supporting Information) and attenuated the effective interacting of SLAMF8 with TREM1 (Figure S2C, Supporting Information). Thereby, we proposed that SLAMF8 might maintain the high protein level of TREM1 via interacting with TREM1, the relative molecular mechanisms are worthy of exploring in the next study.

Monocyte‐macrophage autophagy is linked to inflammation and thrombosis, and its dysfunction is a feature of autoimmune activation.^[^ ^45^ ^]^ In a previous study, impaired autophagy was detected in in a murine APS model.^[^ ^23^ ^]^ In the in vivo and in vitro APS models of our study, we tested downregulated LC3II/I, Beclin‐1, and ATG5 and upregulated p62/SQTM1, indicating a deficiency of autophagy. SLAMF8 or TREM1 knockdown, or OICR‐9429 administration induces autophagy. TREM1 accelerates thrombin generation by inducing TF secretion and is an interesting target for investigating novel inhibitors of thrombotic activity.^[^ ^46^ ^]^ In addition, TREM1 intervention partially compensates for FOXJ2‐ and SLAMF8‐induced TF secretion. These results provide novel evidence that FOXJ2 activation contributes to the pathogenesis of APS by repressing SLAMF8/TREM1‐mediated autophagy and inducing related inflammation and thrombosis.

Recurrent arterial or venous thrombosis is the main symptom of APS. Treatment of APS mainly depends on long‐term anticoagulation therapy to prevent thrombosis. We found that the thrombi of the carotid artery are larger and the blood velocity of the ascending aorta is slower in the in vivo APS model. Significantly, OICR‐9429 administration shrinks the thrombus plaque area, increases the blood velocity, and reduces anti‐β2GPI levels. Meanwhile, FOXJ2‐mediated inflammation is inhibited and autophagy is markedly induced. These results suggested that OICR‐9429 has the potential to prevent inflammation and thrombosis in APS by inactivating FOXJ2/SLAMF8/TREM1 signaling. In order to more accurately explore the role of FOXJ2 in APS, FOXJ2 need to be specifically deleted or overexpressed in monocytes to construct FOXJ2 genetic mouse model, which might be more specific for investigating the function of FOXJ2 in monocytes for regulating APS pathogenesis.

Clinically, aPLs contribute to pregnancy loss, thrombosis, and thrombocytopenia in APS.^[^ ^6^ ^]^ Therefore, we analyzed the correlation between aPLs and FOXJ2 expression and confirmed that FOXJ2 is highly expressed in patients with PAPS and is positively correlated with the positive rate of aPLs. Complement activation is associated with autoimmune disease activity^[^ ^47^, ^48^ ^]^ and emerging evidence has shown low levels of C3 and C4 in patients with PAPS with thrombosis and poor pregnancy outcomes.^[^ ^49^, ^50^ ^]^ Consistent with these findings, we found that aPL‐positive patients with PAPS have lower C3, C4, and PLT levels. Therefore, aPLs may influence APS pathogenesis by affecting the amounts of PLT, complement C3 and C4. Nevertheless, aPL positivity is not significantly related to a history of thrombosis or adverse pregnancy deliveries, and more samples should be collected to explore the relationship between aPL positivity, a history of thrombosis, and APOs.

Taken together, this study is the first to demonstrate that FOXJ2 is highly expressed in the pathogenesis of APS and is related to the presence of aPLs. FOXJ2 directly augments SLAMF8/TREM1 signaling, consequently accelerating APS pathogenesis by repressing autophagy and promoting the release of proinflammatory and prothrombotic cytokines. Furthermore, OICR‐9429 boosts autophagy and blocks inflammation and thrombus formation by downregulating FOXJ2/SLAMF8/TREM1. These findings provide a novel rationale for targeting FOXJ2 using OICR‐9429 and indicate the potential of epigenetic medicine to improve the treatment of APS.

## 4 Experimental Section

### Animal Models of Vascular APS

Female BALB/c mice (8–10 weeks old) were purchased from Beijing Vital River Laboratory Animal Technology. The experimental mice were bred in a specific pathogen‐free (SPF) environment. Mice were randomly divided into three groups (four per group): a) the negative control (NC) group received intraperitoneal injections of bovine serum albumin (BSA) in Freund's adjuvant (F5881, Sigma–Aldrich); b) the β2GPI group received intraperitoneal injections of 100 ug β2GPI protein (11221‐H08H, Sino Biological Inc.) in Freund's adjuvant on days 1, 8, and 14;^[^ ^51^ ^]^ and c) the OICR‐9429‐treated APS (OICR‐9429+β2GPI) group received 5 mg kg^−1^ of OICR‐9429 daily, starting on day 15 and lasting for 7 consecutive days. On day 22, blood samples were collected from the inner canthus to determine the anti‐β2GPI levels, platelet counts (PLT), and activated partial thromboplastin time (APTT). On day 28, Doppler imaging was used to detect the blood velocity in the ascending aorta. Whatman filter paper (3 mm × 1 mm) was soaked with 10% FeCl_3_ and placed under the carotid artery for 5 min. Then, the arteries were removed, and the thrombosis size was measured following hematoxylin‐eosin (HE) staining. In addition, monocytes from thigh bone marrow of mice were extracted using the EasySep Mouse Monocyte Isolation Kit (#19 861, STEMCELL Technologies), and the levels of functional molecules in bone marrow‐derived monocytes were directly examined.

### Participants

Sixty‐four adult patients diagnosed with PAPS according to the Sydney classification criteria between November 2022 and July 2023 were enrolled in this retrospective study.^[^ ^52^ ^]^ Thirty‐two healthy donors (HDs) were selected as the control group and were age‐ and sex‐matched with patients with PAPS. Peripheral blood monocytes were collected from blood samples of patients with PAPS and HDs. Demographic, clinical, and laboratory features were recorded, including age, sex, history of venous or arterial thrombosis and APOs, complement C3 and C4 levels, PLT, normalized silica clotting time (SCT), dilute Russell viper venom time (dRVVT), and titers of aCL and anti‐β2GPI. Antiphospholipid antibodies titers were defined as positive for aCL and anti‐β2GPI titers ≥20 units and for normalized SCT >1.16 and dRVVT >1.11.

### Cell Culture

Human monocyte THP‐1 cells were purchased from the Shanghai Institutes for Biological Sciences (Shanghai, China). The cells were cultured in RPMI 1640 supplemented with 10% fetal bovine serum (FBS; Gibco, USA) and 1% penicillin‐streptomycin (Gibco, USA) and were maintained at 37 °C and 5% CO_2_ in a humidified incubator. Puromycin (Sigma–Aldrich, USA) was used to select stably transfected cells. After starving for 16 h, THP‐1 cells were pretreated with OICR‐9429 (20 µM, HY‐16993, MedChemExpress, USA) for 24 h and stimulated with the immune complex (IC) (β2GPI [100 µg mL^−1^, 11221‐H08H, Sino Biological Inc., China]/anti‐β2GPI [10 µg mL^−1^, 11221‐R003, Sino Biological Inc.]) for 4 h for RNA and DNA analyses and for 6 h for protein analysis.

### Monocytes Isolation

Peripheral blood mononuclear cells were isolated from three HDs using 1.077 g mL^−1^ Lymphoprep density gradient medium (StemCell Technologies, Canada). Monocytes were isolated from peripheral blood mononuclear cells using APC anti‐human CD14 antibody (301 807, Biolegend, USA) and the EasySep Human Monocyte Isolation Kit (#19 359, Stemcell Technologies). The monocyte purity was routinely assessed by flow cytometry. The cell suspension was seeded onto 48‐well plates and allowed to adhere overnight. On the second day, non‐adherent cells were removed and starved for 16 h. Monocytes were pretreated with OICR‐9429 for 24 h and exposed to the IC for 4 h for RNA and DNA analyses and for 6 h for protein analysis.

### Western Blotting

The cells were collected and lysed in RIPA buffer (Beyotime, China) supplemented with 1% phosphatase inhibitor (Epizyme Biotech, China) and 1% protease inhibitor (Epizyme Biotech). The supernatant protein was obtained, and its concentration was measured using a BCA protein quantitative kit (Beyotime). Proteins in the supernatant were separated by SDS‐PAGE, transferred onto a polyvinylidene fluoride (PVDF) membrane, and blocked with 5% non‐fat milk. The protein was incubated with the following primary antibodies at 4 °C overnight: anti‐FOXJ2 (1:500, sc‐514265, Santa Cruz, USA), anti‐SLAMF8 (1:500, MAB21357, Abnova, Taiwan; 1:500, Bioss, bs‐2473R, China), anti‐TREM1 (1:800, 11791‐1‐AP, Proteintech, China), anti‐TLR4 (1:800, ab13556, Abcam), anti‐MyD88 (1:500, sc‐74532, Santa Cruz), anti‐p65 NF‐κB (1:500, sc‐8008, Santa Cruz), anti‐p‐p65 NF‐κB (1:500, sc‐136548, Santa Cruz), anti‐LC3 (1:1000, #4108, CST, USA), anti‐beclin‐1 (1:1000, #3495, CST), anti‐ATG5 (1:1000, #12 994, CST), anti‐p62/SQSTM1 (1:2000, 18420‐1‐AP, Proteintech), and anti‐β‐actin (1:3000, 66009‐1‐Ig, Proteintech). Following incubation with horseradish peroxidase (HRP)‐conjugated goat anti‐mouse IgG H&L (1:5000, ab205719, Abcam) or HRP‐conjugated goat anti‐rabbit IgG H&L (1:5000, ab6721, Abcam) secondary antibodies at room temperature (RT) for 1 h, signals were visualized using an enhanced ECL kit (Millipore, USA).

### Real‐Time Quantitative PCR (RT‐qPCR)

RNA was isolated from cells using the TRIzol reagent (15 596 026, Invitrogen, USA), following the manufacturer's protocols. Total RNA (1 µg) was extracted and reverse transcribed into complementary DNA (cDNA) using Hifair III 1st Strand cDNA Synthesis SuperMix for qPCR (11141ES60, Yeasen Biotec, China). The expression levels of the target genes in the cells were visualized by qPCR using Hieff qPCR SYBR Green Master (11184ES08, Yeasen Biotec). The primers used for qPCR were as follows: FOXJ2 forward‐TACGACAGGCAGAGCAGA, reverse‐AGTCGAAGTCATCAGGGATC; SLAMF8 forward‐TCTCTGCCTGGCACTGGT, reverse‐TGTCTCTGGACCCACTCTGT; TREM1 forward‐GCATCCGCTTGGTGGTGAC, reverse‐ ACATCGGCAGTTGACTTGG; *FOXJ2* promoter forward‐AACTTCACCCCAATCTTCACC, reverse‐ATACCGTCAATTCCAGCTGAC; and *SLAMF8* promoter forward‐CAGATAGTAGTGAGGCAGGCTG; reverse‐GGGCTTACAGGAGCAGATTCTT.

### Enzyme‐Linked Immunosorbent Assay (ELISA)

Cells were seeded onto 24‐well plates (2.5 × 10^5^ cells well^−1^) and treated with different stimuli for 24 h as described above. The levels of IL‐6, IL‐8, TNF‐α, and TF from cell supernatants and mice serum were determined using ELISA kits (Elabscience, China; mlbio China), read at an absorbance of 450 nm, and expressed as pg mL^−1^.

### siRNA Interference

Small interfering RNAs (siRNAs) against SLAMF8 (siSLAMF8#1: GCAACUUCUCCGUGUUGAU; siSLAMF8#2: GAAGGCCUCCUACAAAGAU), TREM1 (siTREM1#1: GACCCUGGAUGUGAAAUGUGA; siTREM1#2: GGCAGAUAAUAAGGGACGGAG), and siNC (negative control) were synthesized by GenePharma Technologies (China). Cells were seeded onto 6‐well plates, and transfection was performed at 50% confluence using jetPRIME in vitro siRNA transfection reagent (#114‐01, Polyplus, Canada) in accordance with the manufacturer's instructions.

### Lentivirus Packaging

Small hairpin RNA (shRNAs) targeting FOXJ2 (shFOXJ2#1: AACCATGACTTTAAATTCTCC; shFOXJ2#2: TCGAACAACTACTACATGTAT) and scrambled negative control (shNC) were synthesized by GeneKai (China). FOXJ2‐overexpression (OE), SLAMF8‐OE, TREM1‐OE, and negative control (vector) were designed and labeled with FLAG. 293T cells (1 × 10^6^) were seeded onto 10 cm‐plates and incubated at 37 °C. When the 293T cells reached 60–70% confluence, they were cotransfected with 20 µg target plasmids, 15 µg pHelper 1.0, and 10 µg pHelper 2.0. After 8 h, the medium was replaced with fresh medium containing 10% FBS. After incubation for 48 and 72 h, the supernatant virus was collected and analyzed for purity and titer.

### Establishing Stable Cells

THP‐1 cells were seeded onto 6‐well plates and transfected with the appropriate virus at 20–30% confluence. After 12 h, the medium containing the virus was replaced with fresh 10% FBS‐RPMI 1640. After 48–72 h, puromycin was applied to select cells for 3–4 days. RT‐qPCR was used to detect knockdown or overexpression efficiencies. Finally, shFOXJ2 and shRNA, FOXJ2‐OE, SLAMF8‐OE, TREM1‐OE, and vector‐transfected stable cells were obtained.

### Chromatin Immunoprecipitation (ChIP)

Cells (1 × 10^7^) were cross‐linked with 1% paraformaldehyde for 10 min at RT and de‐crosslinked in 0.125 m glycine solution for 5 min at RT. Cell lysates were collected and sonicated to obtain soluble chromatin. After incubating with 2 µg anti‐FOXJ2 (sc‐514265, Santa Cruz) or control IgG, immunocomplexes were precipitated to collect DNA. ChIP DNA was sequenced using a BGISEQ‐500 and identified using qPCR. Relative DNA enrichment was represented as ΔCt [normalized IP] = (Ct [IP] − (Ct [input] − log2 [input dilution factor])), input dilution factor = 10, and %Input = 2ˆ(−ΔCt [normalized IP]) × 100%).

### RNA‐Seq

Total RNA from 3 × 10^6^ cells was collected and purified, and RNA products were used to construct sequencing libraries using the NEBNext Ultra RNA Library Prep Kit for Illumina and following manufacturer's protocols. Paired‐end RNA‐Seq was performed using the Illumina NovaSeq 6000 system. Raw reads were trimmed using Trimmomatic to obtain adaptor reads, low‐quality reads, and reads containing N. Trimmed sequence reads were mapped to the UCSC hg38 genome using Hisat2 version 2.0.5. FeatureCounts version 1.5.0‐p3 was used to calculate read counts mapped to the genome. Fragments per kilobase million (FPKM) reads were calculated for each gene. Differentially expressed genes were filtered using the DESeq2 R package (version 1.20.0) and distinguished based on a false discovery rate (FDR) <0.05, and |log_2_FoldChange| >1.

### Luciferase Activity Reporter Assay

The http://jaspar.binf.ku.dk/ website was employed to predict the potential binding of FOXJ2 to the *SLAMF8* promoter. The wild‐type *SLAMF8* promoter (WT‐SLAMF8) was amplified using a luciferase reporter plasmid. A site‐directed mutagenesis kit (Takara, Shiga, Japan) was used to generate mutant *SLAMF8* promoter (Mut‐SLAMF8). WT‐SLAMF8 and Mut‐SLAMF8 were packaged as lentiviruses. THP‐1 cells were cotransfected with WT‐SLAMF8 or Mut‐SLAMF8 lentivirus and FOXJ2‐OE or vector lentivirus. After 48 h, the relative luciferase activity was measured using the dual‐luciferase reporter assay system (E1910, Promega).

### Co‐Immunoprecipitation (Co‐IP)

THP‐1 cells were collected and lysed using cell lysis buffer (P0013, Beyotime). The protein lysate was incubated with protein A+G agarose plus species‐specific IgG (P2055, Beyotime) at 4 °C for 2 h to avoid nonspecific binding. After centrifuging, the protein supernatant was mixed with anti‐FLAG (1:20, ab205606, Abcam) or control IgG (A7001, Beyotime) at 4 °C overnight. Then, protein A+G agarose was added at 4 °C for 3 h. Samples were washed three times with PBS and were ready for western blot assessment.

### Cleavage Under Targets & Tagmentation (CUT&Tag)

The CUT&Tag assay was performed using the Hyperactive Universal CUT&Tag Assay Kit for Illumina (TD903, Vazyme, China). A total of 1 × 10^5^ monocytes or THP‐1 cells were collected and incubated with active ConA beads at RT. After static incubation with the primary antibody at 4 °C overnight and the secondary antibody (1:100) at RT, the ConA bead complex was rotationally incubated with pA/G‐Tnp. DNA extraction beads were used to extract the fragmented DNA, and a DNA spike was added to normalize the sequencing data. The library was constructed using the TruePrepTM Index Kit V2 for Illumina (TD202, Vazyme). VAHTS DNA Clean Beads (#N411, Vazyme) were used to purify the PCR products, and the library concentration and quality were detected using Qubit fluorometric quantification. Finally, the DNA products were subjected to paired‐end Illumina sequencing and qRT‐PCR.

### Assay for Transposase‐Accessible Chromatin with Sequencing (ATAC‐seq)

ATAC‐seq was performed using the TruePrepTM DNA Library Prep Kit V2 for Illumina (TD501, Vazyme, China). Briefly, monocytes or THP‐1 cells (5 × 10^4^) were collected and lysed to collect the cell nuclei. VAHTS DNA Clean Beads (#N411, Vazyme) were used to purify the fragmented DNA, and PCR was performed for library construction using the TruePrepTM Index Kit V2 for Illumina (TD202, Vazyme). After purifying the PCR products, the library concentration and quality were determined by Qubit fluorometric quantification. Finally, paired‐end Illumina sequencing and bioinformatics analyses were performed.

### Transmission Electron Microscopy (TEM)

Autophagosomes were detected via TEM. First, cells (1 × 10^6^) were fixed in PBS (pH 7.4) containing 2.5% glutaraldehyde at RT for 40 min, then at 4 °C overnight. Then, samples were fixed in 1% osmic acid at 4 °C for 1.5 h. After gradient dehydration in 30%, 50%, 70%, 90%, and 100% acetone, specimens were embedded in ethoxyline resin. Samples were sliced into 50 nm sections and stained with 4% uranyl acetate‐lead citrate. Autophagic vacuoles were subsequently photographed using TEM (Philips CM10, Eindhoven, Netherlands).

### Double‐Labeled Immunofluorescence

Cells were fixed in 4% formaldehyde at RT for 30 min and permeabilized with 0.1% Triton X‐100 at 4 °C for 10 min. After saturating with PBS containing 2% BSA at RT for 1 h, the samples were incubated with rabbit polyclonal anti‐SLAMF8 (1:100, bs‐2473R, Bioss, China) and mouse monoclonal anti‐Flag (1:100, ab18230, Abcam), rabbit polyclonal anti‐SLAMF8 (1:100, bs‐2473R, Bioss) and mouse monoclonal anti‐TLR4 (1:100, ab22048, Abcam), or mouse monoclonal anti‐TLR4 (1:100, ab22048, Abcam) and rabbit polyclonal anti‐TREM1 (1:50, 11791‐1‐AP, Proteintech) primary antibodies at 4 °C overnight. Next, samples were incubated with fluorescent goat anti‐mouse IgG H&L (Alexa Fluor 488) (1:300, ab150013, Abcam) and goat anti‐rabbit IgG H&L (Alexa Fluor 555) (1:300, ab150078, Abcam) secondary antibodies at RT in a dark room for 2 h. Subsequently, nuclei were labeled with DAPI (28718‐90‐3, Sigma–Aldrich) by incubation at RT for 5 min. Between all incubation steps, the cells were washed three times with PBS containing 0.2% BSA. Finally, an anti‐fading agent were applied. Fluorescent signals were observed using a confocal microscope (LSM780NLO; Zeiss, Germany).

### Statistical Analysis

Data are presented as mean ± standard error of the mean (SEM) of three independent experiments and were statistically analyzed by a two‐tailed unpaired *t*‐test or Mann‐Whitney U test. The *χ*2‐test was utilized to compare categorical variables. Differences between groups were considered significant at *P < 0.05, **P < 0.01, and ***P < 0.001 and were nonsignificant (ns) at *P* > 0.05. All statistical data were analyzed using GraphPad Prism 7.0.

### Ethics Approval and Consent to Participate

All experiments involving animals were approved by the Ethics Committee of Peking University Third Hospital (Approval Form: 060‐02).

The studies involving human participants were approved by Ethics Committee of Peking University Third Hospital (Approval Form: 053‐01). Written informed consent for participation was not required for this study in accordance with the national legislation and the institutional requirements.

## Conflict of Interest

The authors declare no conflict of interest.

## Author Contributions

Y.T. and L.C. contributed to the study conception and design. The first draft of the manuscript and Figures 1, 2, 3, 4, 5, 6, 7 were prepared by Y.T. The experiments were performed by Y.T., J.Q., and S.Y. Data analysis was performed by H.L. Clinical samples were collected by Q.W., W.F., and Q.L. All authors commented on previous versions of the manuscript and read and approved the final manuscript.

## Supporting information

Supporting Information

Supplemental Data 1

Supplemental Data 2

Supplemental Data 3

## Data Availability

The data that support the findings of this study are available from the corresponding author upon reasonable request.
