# Supplementary material for: H3K4me3‐Mediated FOXJ2/SLAMF8 Axis Aggravates Thrombosis and Inflammation in β2GPI/Anti‐β2GPI‐Treated Monocytes
Source: Adv Sci (Weinh). 2024 Apr 19;11(24):2309140. doi: 10.1002/advs.202309140 (PMC11199983; doi:10.1002/advs.202309140)
Supplement: Supplementary file 1 — Supporting Information [file ADVS-11-2309140-s004.pdf]

ADVANCED SCIENCE
Open Access

Supporting Information

for *Adv. Sci.*, DOI 10.1002/advs.202309140

H3K4me3-Mediated FOXJ2/SLAMF8 Axis Aggravates Thrombosis and Inflammation in β2GPI/Anti-β2GPI-Treated Monocytes

*Yuan Tan, Jiao Qiao, Shuo Yang, Hongchao Liu, Qingchen Wang, Qi Liu, Weimin Feng and Liyan Cui**

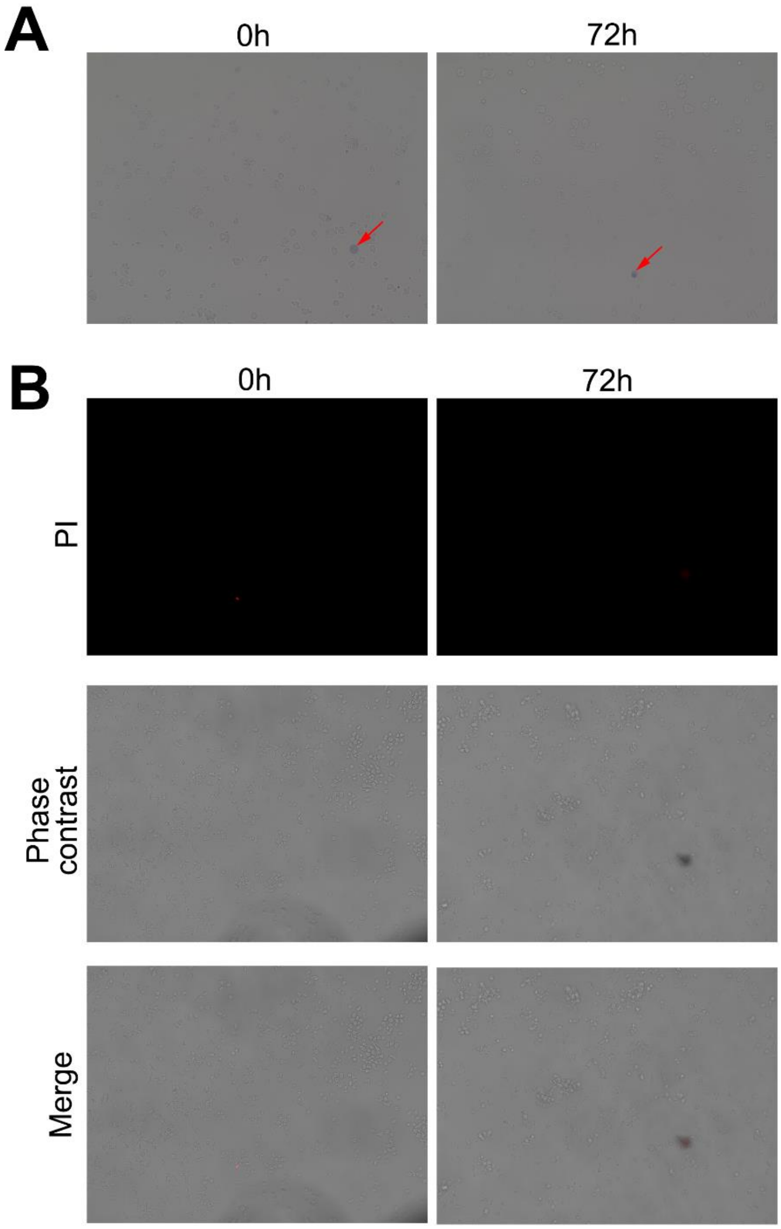

Figure S1. Assessing the viability of monocytes. **A** Assessing the viability of monocytes using trypan blue staining at the beginning (0h) and end (72h) of experiments. Red arrows represented trypan blue-positive cells. **B** Assessing the viability of monocytes using PI staining at the beginning (0h) and end (72h) of the experiment

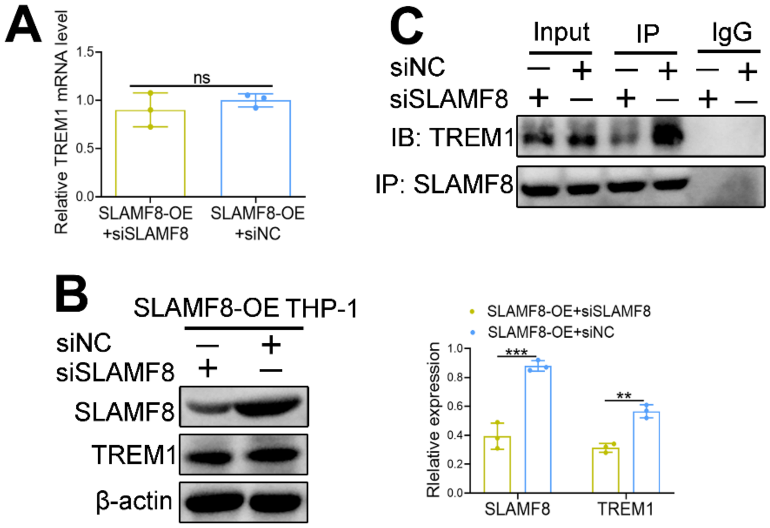

Figure S2. Knockdown of SLAMF8 repressed the interaction of SLAMF8 with TREM1. **A** RT-qPCR detected the mRNA level of TREM1 in SLAMF8-OE+siNC and SLAMF8-OE+siSLAMF8 transfected THP-1 cells. **B** Western blotting detected the protein levels of SLAMF8 and TREM1 in SLAMF8-OE+siNC and SLAMF8-OE+siSLAMF8 transfected THP-1 cells. **C** The interaction of SLAMF8 with TREM1 was measured using co-IP assay in SLAMF8-OE+siNC and SLAMF8-OE+siSLAMF8 transfected THP-1 cells. ns, not significant; $**P < 0.01$; $***P < 0.001$

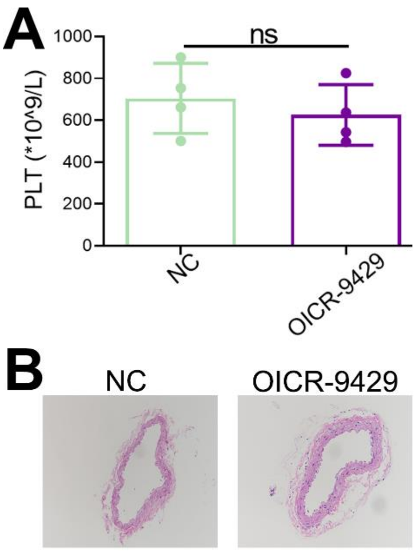

Figure S3. The influence of OICR-9429 on the PLT and the thrombus formation of carotid artery in normal mice. **A** The PLT in NC and OICR-9429 groups. **B** The thrombus formation of carotid artery was validated using HE in NC and OICR-9429 groups. ns, not significant
